# Event and Comment

**Published:** 1920-08

**Authors:** 


### DENTAL REGISTER

THE MAGAZINE OF DENTISTRY

Vol. LXXIV AUGUST, 1920 No. 8

### EVENT AND COMMENT

**Pain and Preventive Dentistry** Probably there is no disease so constantly or universally prevalent as the pain of diseased teeth and their immediate environment. The pain from diseased teeth is not always confined to the teeth themselves but may have wide systemic variations and causal factors. Dental pains often are most real, or they may actually influence the subconscious and sympathetic functions with such intolerable persistence that there is little doubt as to their physical as well as their psychic qualities. Fortunately, the practical outcome is generally beneficent and kindly ministrations are usually practicable.

The pathology of dental diseases is now known because of the exact knowledge we have of the related sciences, but the peculiar anatomical environment leads to very complicated, technical and remedial treatments in many instances, often of a radical character.

Almost every variety of medical procedure has been utilized in the treatment of dental diseases, and in view of the complicated character of diseases, we may congratulate ourselves that we have done so well in relieving dental pains and sufferings.

In the field of obtending pain from dental operations we have practically made most operative procedures painless, especially when the diseased tissues do not seriously complicate the operations. This is made practicable by the use of local and general anesthetics. This has made dental surgery an incalculable factor in the removal of many incurable conditions not practicable with former facilities and methods of treatment. While it is true present methods make no attempt to conserve seriously diseased teeth, systemic contaminations have forced the profession to give more serious attention to dental prophylaxis as the proper remedy for all forms of diseased teeth. Incidentally this has done much to cure or prevent painful conditions of the teeth.

At present, it seems we shall be forced to extract diseased teeth more generally than before to prevent serious focal infections, or learn to take better care of the teeth to prevent infection of the peridental tissues and infection of the pulp through exposure.

Our next serious problem will probably lead us to a closer study of preventive methods for dealing with oral diseases due to infection, which impair the function of the dental tissues and organs. If our present efforts to attain this objective by technical and sanitary influences succeeds, we may also be able to prevent decay of the calcified dental organs, and also do much more than we have heretofore accomplished for the adjacent soft structures through this and other therapeutic activities. It however seems more probable that a well-ordered system of preventive dentistry, based on the ideal development of the deciduous and permanent teeth with prophylactic care, promises more satisfactory results than any treatment of an operation or medical character we have as yet undertaken.

When we are informed that 90% of the people of all ages are suffering with dental caries, and that we are making no well-considered plans for saying the world from this formidable array of painful suffering, it would seem that it was high time we were beginning to try some better means for solving this problem. It might energize us perhaps if we could only visualize the meaning of having a considerable number of young children kept under careful surveillance during the period of their child development, while the deciduous and the adult dentures have been preserved absolutely intact, normal in form and function, to the age of twenty-five years with no pain or disease of serious consequence.

Such highly desirable possibilities may not materialize quickly. Some one has suggested that "a ten-year-old calf can not be grown in a minute," but this does not imply that we should not begin to grow the "superman," should we decide he is practicable. If we are reasonably persistent in our endeavors, and we can increase in knowledge and desire, he may come sooner than we think.

